# The efficacy of pioglitazone for renal protection in diabetic kidney disease

**DOI:** 10.1371/journal.pone.0264129

**Published:** 2022-02-17

**Authors:** Chao-Chung Ho, Yi-Sun Yang, Chien-Ning Huang, Shih-Chang Lo, Yu-Hsun Wang, Edy Kornelius

**Affiliations:** 1 School of Medicine of Chung Shan Medical University, Taichung City, Taiwan; 2 Department of Internal Medicine, Division of Endocrinology and Metabolism, Chung Shan Medical University Hospital, Taichung City, Taiwan; 3 Institute of Medicine of Chung Shan Medical University, Taichung City, Taiwan; 4 Department of Medical Research, Chung Shan Medical University Hospital, Taichung City, Taiwan; University of Colorado Denver School of Medicine, UNITED STATES

## Abstract

There is limited information on the efficacy of pioglitazone in diabetic kidney diseases (DKD). We evaluated whether pioglitazone exerts renal-protective effects in DKD patients. We designed a retrospective cohort study, which included 742 type 2 diabetes mellitus (T2DM) patients with DKD in Taiwan, with eGFR between 30 and 90 ml/min/1.73 m^2^ and UACR level 300–5000 mg/g. Patients not meeting the target range for HbA1c (above 7%) were given additional medication with pioglitazone (n = 111) or received standard care (non-pioglitazone group, n = 631). The primary endpoint was the occurrence of composite renal endpoints, which was defined as sustained eGFR<15 ml/min/1.73 m^2^ (confirmed by two measurements within 90 days); doubling of serum creatinine (compared to baseline); and the presence of hemodialysis or renal transplantation. The median follow-up duration was two years. At baseline, the mean HbA1C levels in the pioglitazone and non-pioglitazone groups were 8.8% and 8.1%, respectively; mean ages were 64.4 and 66.2 years old, respectively; diabetes durations were 14.3 and 12.3 years, respectively. Baseline eGFR showed no significant difference between the pioglitazone and non-pioglitazone groups (55.8 and 58.8 mL/min/1.73 m^2^, respectively). In terms of gender, 63% of patients were male in the pioglitazone group compared with 57% in the non-pioglitazone group. Pioglitazone use did not reduce the risk of composite renal endpoints in DKD patients (HR: 0.97, 95% CI = 0.53–1.77), including persistent eGFR<15 ml/min/1.73 m^2^ (HR = 1.07, 95% CI = 0.46–2.52), doubling of serum creatinine (HR = 0.97, 95% CI = 0.53–1.77), or ESRD (HR = 2.58, 95% CI = 0.29–23.04). The results were not changed after various adjustments. A non-significant albuminuria reduction was also noted after pioglitazone prescription in DKD patients. Further randomized controlled studies are needed to establish the effects of pioglitazone definitively.

## Introduction

Diabetic kidney disease (DKD) is a common microvascular complication of diabetes and is characterized by progressive worsening of albuminuria and decline of kidney function. Patients with DKD have higher risks of end-stage renal disease (ESRD), cardiovascular disease (CVD), and all-cause mortality [1, 2]. The current evidence shows that intensive glucose control, antidiabetic medication, and blood pressure control, especially Renin-Angiotensin-system (RAS) blockade, reduce the risk of kidney disease [3–7].

The role of glucose is still difficult to define in DKD. Still, it appears to involve pivotal intermediates, including oxidative stress and dicarbonyl stress, which promote fibrosis and inflammation in the kidney [8, 9]. Several landmark trials over the past few decades have already shown intensive glucose control in T2DM patients may reduce albuminuria without preventing loss of GFR or progression to ESRD [3, 10, 11]. Nevertheless, the recent introduction of SGLT2 inhibitors (SGLT2i) has given rise to new possibilities in treating DKD. There is growing interest in the efficacy of these antidiabetic agents in terms of hard renal outcomes.

Pioglitazone is an agonist of the peroxisome proliferator-activated receptor γ (PPARγ) and has been widely used in the treatment of type 2 diabetes mellitus (T2DM) for several decades. Many animal studies have demonstrated that PPARγ agonist might potentially reduce the risk of diabetes-induced nephropathy [12–14]. A meta-analysis of human studies determined that pioglitazone may induce robust reductions in the progression of renal disease in patients with or at high risk of T2DM (18.5% reduction of UACR) [15]. In addition, a nationwide cohort study showed Thiazolidinediones, including pioglitazone, reduced the risk of long-term dialysis in advanced DKD patients (HR:0.81, 95% CI = 0.75–0.87) [16]. Another cohort study also revealed the potential benefit of pioglitazone in preserving renal function [17]. The possible mechanisms of protection against diabetic kidney disease include anti-fibrosis, anti-inflammation, inhibition of cell proliferation, and apoptosis [18–22]. However, there are scanty data on the benefits of pioglitazone in terms of hard renal outcomes, especially in severe DKD patients. We hypothesized that the use of pioglitazone could potentially benefit patients with DKD. The CREDENCE trial is a groundbreaking study that tested the primary hypothesis that SGLT2i confers a renal benefit in patients with DKD and the results proved its benefit. As the landmark CREDENCE trial has opened up a new avenue of research on the renal protective function of antidiabetic drugs, we adopted similar enrollment criteria to those used in the trial so that the results of pioglitazone presented in this study could be directly compared with the findings obtained using SGLT2i. Hence, our enrollment criteria were T2DM patients with eGFR 30–90 ml/min/m^2^ and UACR 300–5000 mg/g) [6]. This study aimed to fill the information gap pertaining to renal endpoints in DKD patients with and without a prescription for pioglitazone.

## Materials and methods

### Data resource

We conducted a retrospective study of patients with diabetes who were enrolled in the diabetes shared-care program (DSCP) at Chung Shan Medical University in Taiwan from January 1, 2012, through December 31, 2018 [23]. In brief, The DSCP is a form of pay-for-performance program (P4P) model that is implemented nationally in Taiwan. It is a health care management strategy that links the payment for services to desirable health outcomes. The program emphasizes team care provided by treating physicians, nurses, pharmacists, and dietitians to improve the quality of care for patients with diabetes. The DSCP compensates participating physicians for additional case management fees and desirable health outcomes. The DSCP education model includes appropriate lifestyle modification, encouragement of patients to exercise, frequent self-monitoring of blood sugar, proper medication, and appropriate nutritional intake for diabetes. This study was reviewed and approved by the Institutional Review Board of Chung Shan Medical University Hospital, Taichung, Taiwan, which waived the need for informed consent from participants. This waiver does not affect the rights and welfare of the participants. All experiments were performed in accordance with relevant named guidelines and regulations. Data were analyzed by one independent reviewer. All relevant data are within the manuscript and its S1 Data.

### Study population

**Fig 1:**
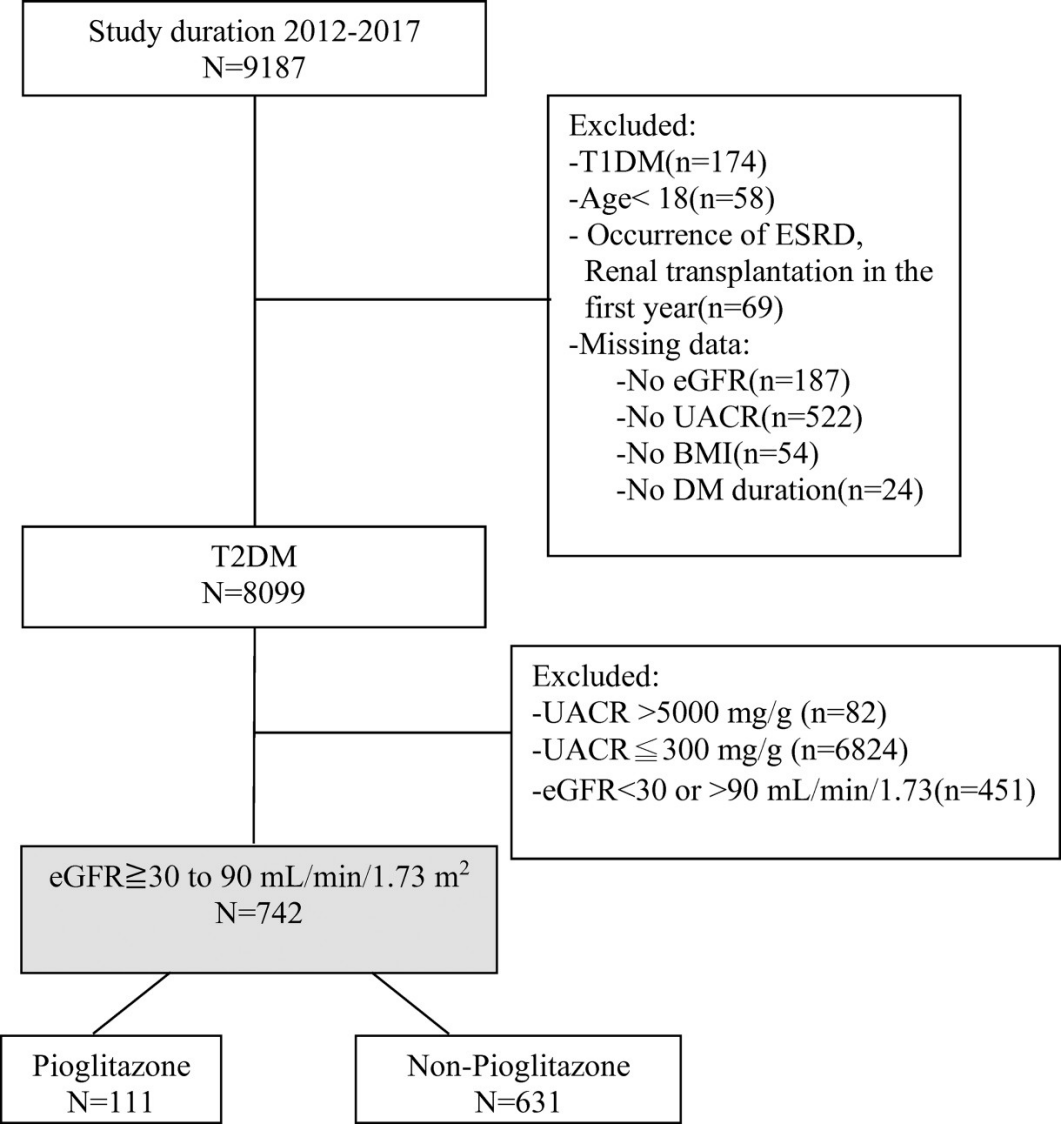
Detailed flow chart of patients’ inclusion and exclusion criteria in this study.

This investigation is a sub-study of our previously published study. The protocol is summarized in brief below [24]. We included patients with T2DM, which was defined as HbA1c ≥ 6.5%. All patients were followed up for at least 365 days. Cohort entry was defined as the earliest HbA1c available in this study. The index date was defined as cohort entry date plus 365 days. The date between cohort entry and index date was defined as the patient selection period. Patients with a pioglitazone prescription during the patient selection period were selected as cases, while patients without a pioglitazone prescription were selected as controls. A detailed study flow chart is shown in Fig 1. The treatment goals in Taiwan are similar to the recommendations of the American Diabetes Association (ADA) [25].

DKD in this study was defined as an average 1-year eGFR (calculated by the modification of diet in renal disease (MDRD) equation) value between 30 and 90 ml/min/1.73 m^2^, in addition to an UACR value between 300 and 5000 mg/g, which were assessed at least three months apart. The study duration was between January 1, 2012, and December 31, 2018.

We excluded patients with eGFR below 30 or more than 90 ml/min/1.73 m^2^, UACR below 300 or more than 5000 mg/g, type 1 DM, history of ESRD, or occurrence of primary renal endpoints within 1 year of follow-up. Patients with missing data such as eGFR, UACR, BMI, or DM duration were also excluded. Comorbidities in this study included a history of hypertension and cardiovascular disease (ICD-9-CM code 410–414, 430–438). The primary endpoint of this study was the occurrence of composite renal endpoints, which was defined as sustained eGFR<15 ml/min/1.73m^2^ (confirmed by two measurements within 90 days); doubling of serum creatinine (compared to baseline); ESRD, including hemodialysis or renal transplantation; or the study end date (December 31, 2018).

### Statistical analysis

Descriptive statistical analyses, including the chi-squared test and Student’s t-test, were used to evaluate categorical and continuous variables. Cox proportional hazards models were used to compare the composite renal outcomes between pioglitazone users and non-pioglitazone users. The analysis of the effects of pioglitazone was further adjusted for all variables, including age, gender, BMI, HbA1c, DM duration, oral antidiabetic medication, hypertension, and CVD. The results are presented as hazard ratios (HRs) with 95% confidence intervals (CIs). We compared the cumulative incidence of composite renal outcomes over time between pioglitazone users and non-pioglitazone users using the Kaplan–Meier method and log-rank test. A two-tailed p-value of <0.05 was considered significant. All analyses were conducted using the SPSS statistical package, version 18 (SPSS; Chicago, Illinois).

## Results

**Fig 2:**
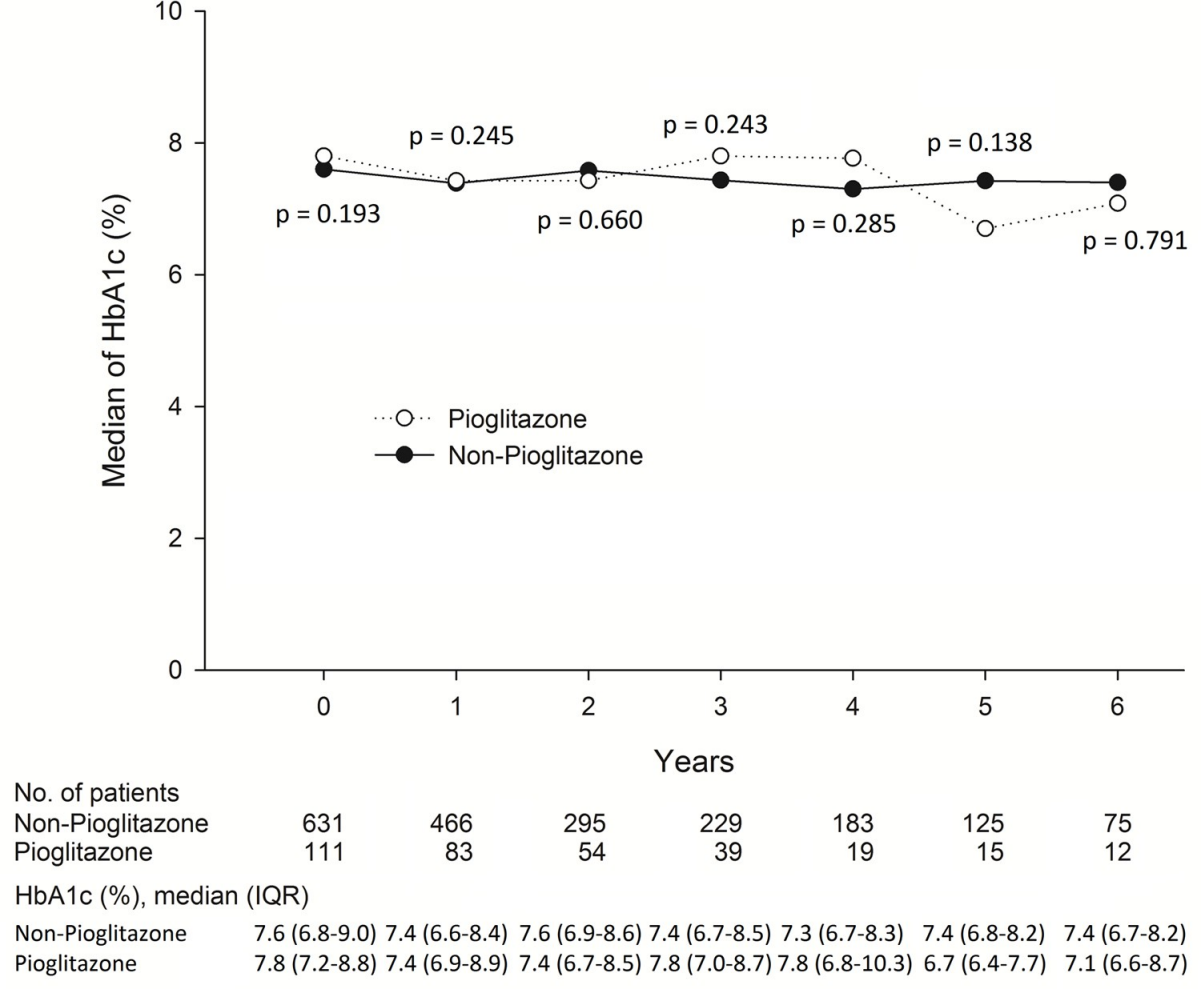
Association between HBA1c between Pioglitazone and non-Pioglitazone users.

**Fig 3:**
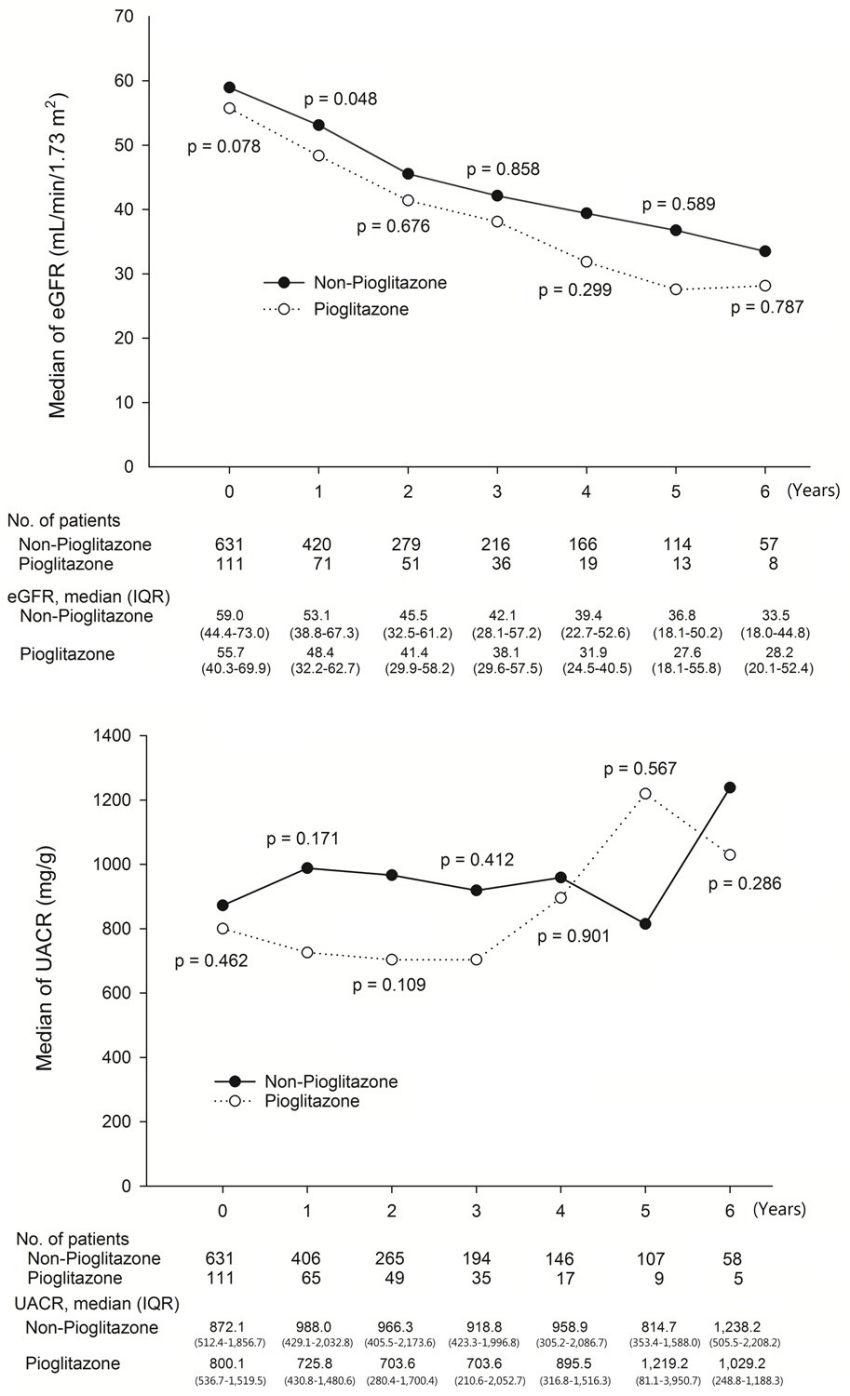
Association between eGFR and UACR between Pioglitazone and non- Pioglitazone users.

We enrolled 742 DKD patients in this study. The baseline characteristics of the pioglitazone and non-pioglitazone groups were generally similar. The mean follow-up period was 2.2 ± 1.8 and 2.9 ± 1.9 years in the pioglitazone and non-pioglitazone groups, respectively (Table 1). The mean HbA1C values were 8.8% ± 5.9 and 8.1% ± 2.5, respectively. During the observation period, both HbA1_C_ and renal function in the pioglitazone and non-pioglitazone groups were similar (Fig 2), which indicated there were no obvious differences in blood glucose between the two study groups. However, despite the lack of a significant difference, the pioglitazone group had a mild better UACR compared to that of the non-pioglitazone group during the study period (Fig 3).

**Table 1:** Baseline demographic characteristics of patients.

	Pioglitazone N = 111	Non-Pioglitazone N = 631	
	n (%)	n (%)	p value
HbA1c, M ± SD	8.8 ± 5.9	8.1 ± 2.5	0.214
Age (index date)			0.905
<55	20 (18)	103 (16.3)	
55–65	33 (29.7)	190 (30.1)	
≧65	58 (52.3)	338 (53.6)	
M ± SD	64.4 ± 11.2	66.2 ± 12.1	0.138
Gender			0.249
Female	41 (36.9)	270 (42.8)	
Male	70 (63.1)	361 (57.2)	
BMI^†^			0.418
<18.5	0 (0)	9 (1.4)	
18.5–23.9	45 (40.5)	264 (41.8)	
≧24	66 (59.5)	358 (56.7)	
DM duration (year)			0.135
<5	7 (6.3)	78 (12.4)	
5–10	36 (32.4)	213 (33.8)	
≧10	68 (61.3)	340 (53.9)	
M ± SD	14.3 ± 7.8	12.3 ± 7.5	0.011
eGFR			
30–45	39(35.1)	162(25.7)	
45–60	26(23.4)	163(25.8)	
60–90	46(41.4)	306(48.5)	
M ± SD	55.8 ± 17.5	58.8 ± 16.6	0.084
UACR			
<1000	64(57.7)	351(55.6)	
≧1000	47(42.3)	280(44.4)	
M ± SD	1209.3 ± 1017.2	1317.4 ± 1075.1	0.325
AGI	1 (0.9)	8 (1.3)	1
DPP4i	65 (58.6)	343 (54.4)	0.412
Meglitinide	20 (18)	61 (9.7)	0.009
GLP1	3 (2.7)	13 (2.1)	0.720
Glucophage	89 (80.2)	416 (65.9)	0.003
Insulin	42 (37.8)	246 (39)	0.819
SGLT2i	22 (19.8)	22 (3.5)	<0.001
Sulfonylurea	53 (47.7)	264 (41.8)	0.246
ARB/ACEI	65 (58.6)	340 (53.9)	0.362
Statin	56 (50.5)	344 (54.5)	0.428
Hypertension	76 (68.5)	423 (67)	0.767
History of CVD	7 (6.3)	77 (12.2)	0.071

Abbreviations: BMI: body mass index; AGI: α- glucosidase inhibitor DPP4i: dipeptidyl peptidase IV inhibitor; GLP1: glucagon like peptide 1 agonist; SGLT2i: sodium-glucose cotransporter-2 inhibitor; ARB/ACEI: angiotensin receptor blocker/ angiotensin-converting enzyme inhibitor; CVD: cardiovascular disease; eGFR: estimated glomerular filtration rate; UACR: urine albumin to creatinine ratio

Table 2 shows that the use of pioglitazone did not reduce the risk of composite renal endpoint (HR: 0.97, 95% CI 0.53–1.77). To evaluate the dose-response, we further divided pioglitazone users into three groups. However, higher pioglitazone dose did not confer any significantly greater renal benefit compared to the control group. Moreover, the result was not changed after adjustment for the baseline characteristics, medication, and comorbidities. The average HbA1C over the entire duration of the study period was also included in the model. Multivariate Cox analysis demonstrated a non-significant benefit of pioglitazone in DKD patients with an HR of 0.95 (95% CI = 0.52–1.76).

**Table 2:** The occurrence of primary renal endpoint between Pioglitazone and non- Pioglitazone patients.

	N	No. of renal disease	Observed Person-Years	Incidence density (Per 1000 Person-Years)	Crude HR	95% C.I.	Adjusted HR†	95% C.I.
Pioglitazone								
No	631	93	1807	51.5	1		1	
Yes	111	12	248	48.4	0.97	0.53–1.77	0.95	0.52–1.76
Cumulative dose of pioglitazone								
No	631	93	1806.70	51.5	1		1	
<60 mg	34	4	94.90	42.1	0.84	0.31–2.28	0.72	0.26–1.97
60–1700 mg	37	6	98.62	60.8	1.15	0.50–2.63	1.45	0.61–3.41
≥1700 mg	40	2	54.42	36.8	0.83	0.20–3.40	0.78	0.19–3.25

†Adjusted for age, gender, body mass index, HbA1c, diabetes duration, medication, and comorbidities

**Fig 4:**
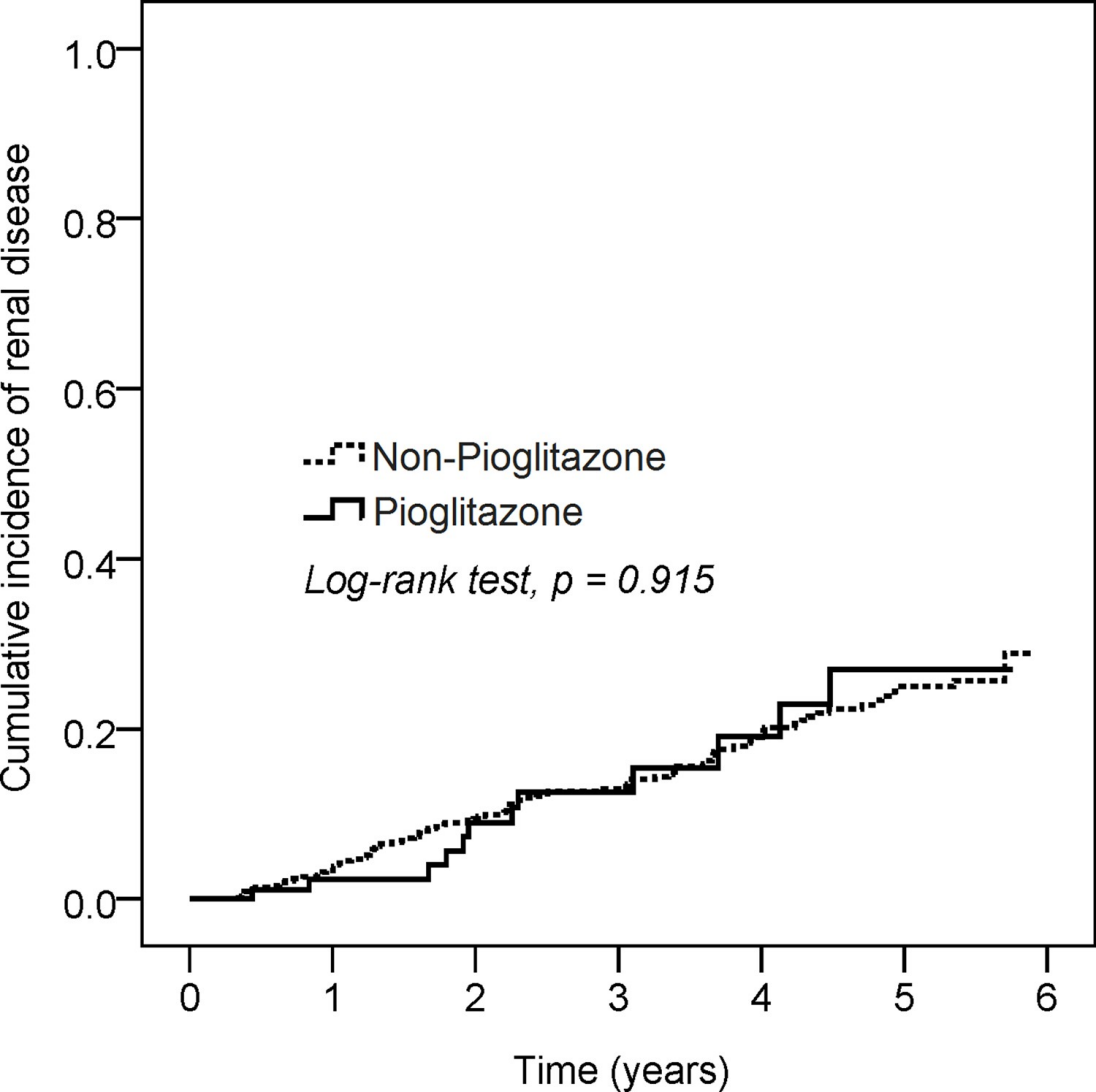
Kaplan-Meier analysis of renal disease between Pioglitazone and non- Pioglitazone users.

Kaplan-Meier analysis (Fig 4) showed that by the end of the follow-up period, the cumulative incidence of renal disease in the two cohorts was similar (log-rank test: P = 0.915). Table 3 shows the subgroup analyses of composite renal outcomes using a Cox proportional hazard model. The following variables were included in the model: age, sex, body mass index (BMI), HbA1c, diabetes duration, antidiabetic agents, RAS blockade, statin, history of cardiovascular disease, level of eGFR, and UACR. The addition of pioglitazone to the analysis of the aforementioned variables did not reduce the risk of composite renal diseases in DKD patients. Table 4 shows a comparison of individual renal endpoints between pioglitazone and non-pioglitazone users. There were no significant differences in the risks of composite renal outcomes, including persistent eGFR<15 ml/min/1.73 m^2^ (HR = 1.01, 95% CI = 0.42–2.4), doubling of serum creatinine (HR = 0.96, 95% CI = 0.52–1.77), or ESRD (HR = 0.57, 95%, CI = 0.003–96.53). Further sensitivity analysis for declines in eGFR of 30%, 40%, and 50% was performed and also revealed no significant differences between pioglitazone and non-pioglitazone users (S1 Table). In addition, “regression of albuminuria” was also calculated, which was defined as a reduction in UACR from baseline (300–5000 ml/min/1.73 m^2^) to less than 300 ml/min/1.73 m^2^ after treatment with pioglitazone. The results shown in S2 Table revealed no statistical difference between pioglitazone and non-pioglitazone users.

**Table 3:** Subgroup analysis of renal outcomes between Pioglitazone users and non-Pioglitazone users.

	Pioglitazone		Non-Pioglitazone			
	N	No. of renal disease	N	No. of renal disease	HR	95% CI
Age						
<55	20	3	103	24	0.92	0.28–3.08
55–65	33	2	190	29	0.54	0.13–2.28
≧65	58	7	338	40	1.32	0.59–2.95
Gender						
Female	41	5	270	40	1.17	0.46–2.98
Male	70	7	361	53	0.87	0.39–1.91
BMI						
18.5–23.9	45	5	264	25	1.52	0.58–3.99
<18.5 or ≧24	66	7	367	68	0.77	0.35–1.69
HbA1c						
<7%	24	3	184	19	1.12	0.33–3.80
7–9%	63	4	298	43	0.61	0.22–1.71
>9%	24	5	149	31	1.62	0.63–4.19
DM duration (year)						
<5	7	1	78	12	1.32	0.17–10.21
5–10	36	3	213	21	1.47	0.44–4.96
≧10	68	8	340	60	0.83	0.40–1.75
DPP4 inhibitors						
No	46	6	288	44	1.18	0.50–2.77
Yes	65	6	343	49	0.81	0.34–1.88
Meglitinide						
No	91	9	570	84	0.90	0.45–1.80
Yes	20	3	61	9	1.37	0.36–5.19
GLP1						
No	108	12	618	92	0.98	0.54–1.79
Yes	3	0	13	1	NA	NA
Glucophage						
No	22	4	215	35	1.02	0.36–2.88
Yes	89	8	416	58	0.94	0.45–1.98
Insulin						
No	69	7	385	42	1.14	0.51–2.55
Yes	42	5	246	51	0.84	0.33–2.11
SGLT2						
No	89	12	609	93	1.01	0.55–1.84
Yes	22	0	22	0	NA	NA
Sulfonylurea						
No	58	4	367	48	0.67	0.24–1.87
Yes	53	8	264	45	1.26	0.59–2.68
ARB/ACEI						
No	46	5	291	39	0.97	0.38–2.46
Yes	65	7	340	54	0.97	0.44–2.13
Statin						
No	55	6	287	46	1.00	0.43–2.35
Yes	56	6	344	47	0.92	0.39–2.15
CVD						
No	104	11	554	76	0.99	0.53–1.87
Yes	7	1	77	17	0.72	0.10–5.41
eGFR						
30–45	39	5	162	37	0.54	0.21–1.38
45–60	26	2	163	24	0.80	0.19–3.38
60–90	46	5	306	32	1.58	0.61–4.08
UACR						
<1000	64	3	351	26	0.84	0.25–2.79
≧1000	47	9	280	67	1.03	0.51–2.06

Abbreviations: BMI: body mass index; DPP4i: dipeptidyl peptidase IV inhibitor; GLP1: glucagon like peptide 1 agonist; SGLT2i: sodium-glucose cotransporter-2 inhibitor; ARB/ACEI: angiotensin receptor blocker/ angiotensin-converting enzyme inhibitor; CVD: cardiovascular disease; eGFR: estimated glomerular filtration rate; UACR: urine albumin to creatinine ratio

NA: not applicable

**Table 4:** Risk of primary renal outcome in patients taking Pioglitazone based on Cox proportional hazard model.

	No. of renal disease	Observed Person-Years	Incidence Density (Per 1000 Person-Years)	Crude HR	95% C.I.	Adjusted HR	95% C.I.
Persistent GFR <15^†^							
Pioglitazone							
No	46	1922	23.9	1		1	
Yes	6	254	23.6	1.07	0.46–2.52	1.01	0.42–2.4
Creatinine doubling^†^							
Pioglitazone							
No	93	1809	51.4	1		1	
Yes	12	248	48.4	0.97	0.53–1.77	0.96	0.52–1.77
ESRD^†^^.^							
Pioglitazone							
No	4	2030.38	2.0	1		1	
Yes	1	264.33	3.8	2.58	0.29–23.04	0.57	0.003–96.53

†Adjusted for age, gender, BMI, DM duration, medication, and comorbidities.

## Discussion

In this retrospective cohort study, although the use of pioglitazone was associated with albuminuria reduction, we found that patients with DKD who received pioglitazone did not have better composite hard renal outcomes compared to non-pioglitazone users. The composite renal outcomes included doubling of serum creatinine level, persistent low eGFR (<15 ml/min/1.73m^2^), and ESRD.

Pioglitazone functions principally as an agonist of PPARγ and partially as an activator of PPARα. It is used in the treatment of T2DM and has already proved to be effective in improving insulin sensitivity, hyperglycemia, and lipid metabolism. The PROactive trial was a landmark trial designed to investigate the effectiveness and safety of pioglitazone in diabetic patients. The results showed a reduction of all-cause mortality, non-fatal myocardial infarction, and stroke in the intervention group [26]. The clinical trial did not investigate renal outcomes in the setting of diabetic kidney disease. Consequently, further research in this area is warranted, particularly as PPARγ is widely expressed in the kidney and may therefore play an essential role in renal function [27].

Although several adverse effects, such as weight gain, edema, and fluid retention leading to congestive cardiac heart failure, were reported in users of thiazolidinediones (TZDs) [3, 26, 28]. The effects of TZDs on cardiovascular events and mortality remain unclear [29, 30]. The conflicting results may be due to the inclusion of different TZDs in the studies. The mechanisms underlying the apparent differences in cardiovascular risk and mortality between rosiglitazone and pioglitazone have not been clearly elucidated, but one possible explanation is that pioglitazone elevated HDL levels and reduced TG levels, while rosiglitazone increased LDL and TC levels [31]. It is also worth noting that recent studies demonstrated that pioglitazone reduced the risk of stroke and myocardial infarction in non-diabetic, insulin-resistant patients with a history of stroke or transient ischemic attack [32, 33]. The results of the aforementioned studies on pioglitazone are encouraging, and it is therefore reasonable to further evaluate its efficacy with respect to renal endpoints.

In addition, several clinical studies have already confirmed TZDs can reduce urinary albumin excretion and proteinuria in DKD patients. A meta-analysis involving 2860 patients with diabetes demonstrated that TZD significantly decreased levels of urinary albumin [13]. Another meta-analysis, which included 26 studies with 19645 participants, also showed pioglitazone reduced albuminuria by 18.5% (95% CI = 21.1–16.0) in patients with or at high risk of T2DM [15]. The potential reno-protective mechanism of pioglitazone is not completely understood, but it might involve certain properties related to anti-apoptosis [22], anti-inflammation [20], and repression of transforming growth factor-*β* (TGF-*β*) pathways in renal interstitial fibroblasts, which play an essential role in kidney fibrosis [21].

While there is evidence showing that TZD may benefit renal functions, as mentioned above, pioglitazone has never been shown to improve hard renal outcomes in a clinical study. RAS blockade is the first medication that has been proven to have a reno-protective effect in diabetic patients [4]. However, some research has demonstrated the use of RAS blockers in advanced CKD patients (stage 4 or 5) may accelerate progression to ESRD without enhancing survival [34]. As a result, there is an urgent need to find new therapeutic medications for DKD patients to slow down the progression of kidney disease to ESRD. The CREDENCE and DAPA-CKD trials have yielded groundbreaking results demonstrating that the new class of antidiabetic agents, known as sodium-glucose co-transporter 2 inhibitors (SGLT2-i), were associated with significantly better kidney endpoints in a DKD population [6, 7]. The possible mechanisms underlying the beneficial effects of SGLT2-i on the kidney include reduced tubular workload, mitigation of hypoxia in the proximal tubule, restoration of tubuloglomerular feedback, and diuretic effects, as well as anti-inflammatory and antifibrotic effects [35]. Taken together, the promising results of recent research on SGLT2-i, in addition to the aforementioned studies and the ADVANCE trial, which showed a 21% relative reduction in nephropathy with intensive glucose control [3], indicate that pioglitazone may have similar reno-protective effects.

In order to directly compare hard renal outcomes between pioglitazone users and SGLT2-i users, our enrollment criteria were similar to those used in the CREDENCE trials (T2DM patients with eGFR 30–90 ml/min/m^2^ and UACR 300–5000 mg/g). In our study, although pioglitazone use was associated with mild albuminuria reduction, it did not provide any additional benefit based on composite hard renal endpoints. In the subgroup analysis, we further analyzed 201 patients with more severe DKD, i.e., eGFR<45 ml/min/1.73m^2^, which also demonstrated pioglitazone conferred no additional benefit or harm. Nevertheless, although the sample size was small due to the limited use of SGLT2 inhibitors in this study population, it is worth noting that in our data, as shown in Table 3, there was no progression of renal disease in any of the patients who received SGLT2 inhibitors. This result is consistent with a previous study that found combination therapy of SGLT2 inhibitor and PPARγ agonists might be an ideal agent in clinical practice [36].

In our opinion, the major reason why pioglitazone did not improve renal endpoints in more severe DKD patients is that the mean follow-up duration of our study was relatively short. However, it is worth noting that the CREDENCE trial was stopped early (the median follow-up was 2.6 years) because an interim analysis found evidence of a clear benefit [6].

This study has several strengths. Firstly, this investigation evaluated the efficacy of pioglitazone with respect to hard renal outcomes, which have largely not been discussed in previous studies on pioglitazone. Secondly, although we cannot directly compare our results to the findings of randomized control trials, our enrollment criteria were similar to those of the CREDENCE trial, which allowed direct comparisons of two different antidiabetics. Thirdly, complete laboratory data, including HbA1C, UACR, and eGFR, were obtained from our cohort, and these data clearly possess clinical value in real-world practice. There were also some limitations in this study that should be acknowledged. Firstly, this was a retrospective observational study, which might have introduced potential selection bias. Secondly, we were unable to assess the patients’ physical activity and lifestyle factors, which might have confounded the composite renal outcomes. Thirdly, the sample size was relatively small, and all patients were from a single hospital in Taiwan. Fourthly, medication compliance could not be determined in this study as the analyses were conducted using medical prescription records.

## Conclusion

Pioglitazone did not reduce the risk of composite renal endpoints in DKD patients. Further randomized controlled studies are needed to definitively establish the effects of pioglitazone.

## Supporting information

S1 Table The occurrence of eGFR decline between Pioglitazone and non-Pioglitazone patients. (DOCX)

S2 Table The occurrence of regression of UACR between Pioglitazone and non-Pioglitazone users. (DOCX)

S1 Data (XLSX)

## Abbreviations

AGI: α- glucosidase inhibitor
ARB/ACEI: angiotensin receptor blocker/angiotensin-converting enzyme inhibitor
BMI: body mass index
CVD: Cardiovascular disease
DPP4i: dipeptidyl peptidase IV inhibitor
DKD: Diabetic kidney disease
eGFR: estimated glomerular filtration rate
ESRD: End-stage renal disease
GLP-1 RA: glucagon-like peptide 1 receptor agonist
PPAR: peroxisome proliferator-activated receptor
SGLT2i: sodium-glucose cotransporter-2 inhibitor
UACR: urine albumin to creatinine ratio
