# Supplementary material for: The efficacy of pioglitazone for renal protection in diabetic kidney disease
Source: PLoS One. 2022 Feb 17;17(2):e0264129. doi: 10.1371/journal.pone.0264129 (PMC8853567; doi:10.1371/journal.pone.0264129)
Supplement: S1 Table — (DOCX) [file pone.0264129.s001.docx]

**S1 Table** . The occurrence of eGFR decline between Pioglitazone and non-Pioglitazone patients

|  | N | No. of renal disease | Crude HR | 95% C.I. | Adjusted HR^†^ | 95% C.I. |
| --- | --- | --- | --- | --- | --- | --- |
| **GFR decline 30%** |  |  |  |  |  |  |
| Pioglitazone |  |  |  |  |  |  |
| No | 631 | 218 | 1 |  | 1 |  |
| Yes | 111 | 34 | 1.06 | 0.74-1.53 | 0.98 | 0.68-1.42 |
| Cumulative dose of pioglitazone |  |  |  |  |  |  |
| No | 631 | 218 | 1 |  | 1 |  |
| <60 mg | 34 | 12 | 1.04 | 0.58-1.86 | 0.91 | 0.5-1.64 |
| 60-1700 mg | 37 | 14 | 1.16 | 0.68-1.99 | 1.16 | 0.67-2.02 |
| ≥1700 mg | 40 | 8 | 0.96 | 0.47-1.95 | 0.86 | 0.42-1.77 |
| **GFR decline 40%** |  |  |  |  |  |  |
| Pioglitazone |  |  |  |  |  |  |
| No | 631 | 161 | 1 |  | 1 |  |
| Yes | 111 | 20 | 0.88 | 0.55-1.39 | 0.82 | 0.51-1.32 |
| Cumulative dose of pioglitazone |  |  |  |  |  |  |
| No | 631 | 161 | 1 |  | 1 |  |
| <60 mg | 34 | 7 | 0.81 | 0.38-1.74 | 0.71 | 0.33-1.54 |
| 60-1700 mg | 37 | 9 | 1.00 | 0.51-1.95 | 1.06 | 0.53-2.1 |
| ≥1700 mg | 40 | 4 | 0.76 | 0.28-2.07 | 0.65 | 0.24-1.8 |
| **GFR decline 50%** |  |  |  |  |  |  |
| Pioglitazone |  |  |  |  |  |  |
| No | 631 | 124 | 1 |  | 1 |  |
| Yes | 111 | 17 | 1.05 | 0.63-1.74 | 1.02 | 0.61-1.72 |
| Cumulative dose of pioglitazone |  |  |  |  |  |  |
| No | 631 | 124 | 1 |  | 1 |  |
| <60 mg | 34 | 5 | 0.81 | 0.33-1.97 | 0.72 | 0.29-1.79 |
| 60-1700 mg | 37 | 8 | 1.19 | 0.58-2.42 | 1.30 | 0.63-2.72 |
| ≥1700 mg | 40 | 4 | 1.23 | 0.45-3.37 | 1.12 | 0.4-3.11 |

†Adjusted for age, gender, body mass index, HbA1c, diabetes duration, medication, and comorbidities.
