# Supplementary material for: The efficacy of pioglitazone for renal protection in diabetic kidney disease
Source: PLoS One. 2022 Feb 17;17(2):e0264129. doi: 10.1371/journal.pone.0264129 (PMC8853567; doi:10.1371/journal.pone.0264129)
Supplement: S2 Table — (DOCX) [file pone.0264129.s002.docx]

**S2 Table** . The occurrence of regression of UACR between Pioglitazone and non-Pioglitazone users

| **Outcome: UACR<300** |  |  |  |  |  |  |
| --- | --- | --- | --- | --- | --- | --- |
| Pioglitazone |  |  |  |  |  |  |
| No | 631 | 144 | 1 |  | 1 |  |
| Yes | 111 | 24 | 1.09 | 0.71-1.68 | 1.06 | 0.68-1.65 |
| Cumulative dose of pioglitazone |  |  |  |  |  |  |
| No | 631 | 144 | 1 |  | 1 |  |
| <60 mg | 34 | 10 | 1.36 | 0.72-2.59 | 1.39 | 0.72-2.68 |
| 60-1700 mg | 37 | 8 | 1.01 | 0.49-2.05 | 0.95 | 0.46-1.95 |
| ≥1700 mg | 40 | 6 | 0.88 | 0.39-2.01 | 0.86 | 0.37-1.98 |

†Adjusted for age, gender, body mass index, HbA1c, diabetes duration, medication, and comorbidities.
